# Supplementary material for: The Communication Satisfaction of Geriatric Patients Treated by Dental Students and Dentists in a University Dental Clinic: A Cross-Sectional Study
Source: Geriatrics (Basel). 2025 Jul 14;10(4):93. doi: 10.3390/geriatrics10040093 (PMC12286155; doi:10.3390/geriatrics10040093)
Supplement: Supplementary file 1 [file geriatrics-10-00093-s001.zip › geriatrics-3546271-supplementary.pdf]

QUESTIONÁRIO

O presente questionário tem como objetivo avaliar a satisfação de pacientes geriátricos relativamente à comunicação com os alunos pré-graduados do curso de Medicina Dentária da Universidade Egas Moniz School of Health & Science ou médicos dentistas.

Neste questionário não há respostas certas ou erradas.

Este inquérito é ANÓNIMO e CONFIDENCIAL. NÃO ASSINAR.

Atendido por:

☐ Alunos pré-graduados

☐ Médicos Dentistas

Parte I: Dados Sociodemográficos

1. Idade:____

2. Sexo Biológico:

☐ Homem ☐ Mulher

3. Nacionalidade:

☐ Portugal

☐ Outro: ______________________

4. Português é o seu idioma principal?

☐ Sim

☐ Não. Qual?____

5. Estado Civil:

☐Solteiro

☐Casado ou a viver em união de facto

☐Divorciado ou separado

☐Viúvo

6. Tem filhos?

☐Sim. Quantos? _____________________________

Idade: ________________________________

☐Não

7. Vive atualmente sozinho ou acompanhado?

☐ Sozinho

☐ Acompanhado. De quem? ____________________________

Anexo III – Questionário.

8. Área de residência:

☐ Urbano

☐ Rural

9. Habilitações literárias:

☐ Até 4º ano

☐ 5º a 9º ano

☐ Até 12º ano

☐ Licenciatura

☐ Mestrado

☐ Doutoramento

☐ Cursos Técnicos Superiores Profissionais (CTeSP)

10. Qual o seu regime de trabalho? ____________________

☐Reformado

☐Trabalhando em tempo integral

☐Trabalhando em meio período

☐Desempregado

☐Outro (especifique)________________

11. Número de contactos que teve com os profissionais de saúde desta instituição nos últimos 12 meses:

☐1

☐2

☐3 ou + _______

Parte II: Informações sobre Saúde Geral do Utente

1. Motivo da consulta?

____

____

2. Possui algum diagnóstico de doença crónica ou cognitiva?

☐ Sim. Qual? ____

☐ Não

3. Tem problemas de audição?

☐Sim. Qual? ____

☐Não

4. **Tem problemas na fala?**

☐ Sim. Qual?____

☐ Não

5. **Tem problemas de visão?**

☐ Sim. Qual?____

☐ Não

Parte III: Informações sobre a Satisfação dos Utentes Geriátricos

1= Muito Insatisfeito;<br>2= Insatisfeito;<br>3= Nem Satisfeito nem Insatisfeito;<br>4= Satisfeito;<br>5= Muito Satisfeito;<br>N/A= Não Aplicável

I- Avalie a sua satisfação na COMUNICAÇÃO VERBAL COM A EQUIPA DE SAÚDE em relação aos seguintes itens:

1. Explicação dada ao seu problema de saúde:
   ☐1 ☐2 ☐3 ☐4 ☐5 ☐N/A

2. Explicação do seu plano de tratamento/intervenção:
   ☐1 ☐2 ☐3 ☐4 ☐5 ☐N/A

3. Informação como vai tomar a medicação:
   ☐1 ☐2 ☐3 ☐4 ☐5 ☐N/A

4. Comunicação de quando tem um novo exame/tratamento ou consulta:
   ☐1 ☐2 ☐3 ☐4 ☐5 ☐N/A

5. Linguagem acessível:
   ☐1 ☐2 ☐3 ☐4 ☐5 ☐N/A

6. Explicação do exame/tratamento ou intervenção a que estavam a submeter:
   ☐1 ☐2 ☐3 ☐4 ☐5 ☐N/A

II- Classifique a sua SATISFAÇÃO GLOBAL em relação à comunicação não verbal:

☐1 ☐2 ☐3 ☐4 ☐5

III- Avalie a sua satisfação na COMUNICAÇÃO NÃO VERBAL com a equipa de saúde em relação aos seguintes itens:

1. Contacto visual quando falaram consigo:
   ☐1 ☐2 ☐3 ☐4 ☐5 ☐N/A

2. Atenção dispensada:
   ☐1 ☐2 ☐3 ☐4 ☐5 ☐N/A

3. Concordância dos gestos e expressões da equipa de saúde com o que diziam:
   ☐1 ☐2 ☐3 ☐4 ☐5 ☐N/A

4. Apoio da equipe de saúde:
   ☐1 ☐2 ☐3 ☐4 ☐5 ☐N/A

IV- Classifique a sua SATISFAÇÃO GLOBAL em relação à comunicação não verbal:

☐1 ☐2 ☐3 ☐4 ☐5

V- Avalie a sua satisfação na EMPATIA com a comunicação da equipe de saúde em relação aos seguintes itens:

1. Simpatia:
   ☐1 ☐2 ☐3 ☐4 ☐5 ☐N/A

2. Empatia (profissionais colocaram-se no seu lugar):
   ☐1 ☐2 ☐3 ☐4 ☐5 ☐N/A

3. Cuidado dispensado pelos profissionais de saúde:
   ☐1 ☐2 ☐3 ☐4 ☐5 ☐N/A

4. Compreensão:
   ☐1 ☐2 ☐3 ☐4 ☐5 ☐N/A

VI- Classifique a sua SATISFAÇÃO GLOBAL em relação a empatia:

☐1 ☐2 ☐3 ☐4 ☐5

VII- Avalie a sua satisfação no RESPEITO com a comunicação da equipa em relação aos seguintes itens:

1. Tratamento personalizado (pelo nome) :
   ☐1 ☐2 ☐3 ☐4 ☐5 ☐N/A

2. Respeito (quando falou dos seus problemas de saúde):
☐1 ☐2 ☐3 ☐4 ☐5 ☐N/A

3. Resposta (às suas sobre o seu problema de saúde):
☐1 ☐2 ☐3 ☐4 ☐5 ☐N/A

4. Respeito pela sua intimidade:
☐1 ☐2 ☐3 ☐4 ☐5 ☐N/A

VIII- Classifique a sua SATISFAÇÃO GLOBAL em relação aos respeito na comunicação:
☐1 ☐2 ☐3 ☐4 ☐5

IX- Avalie a sua satisfação na RESOLUÇÃO DE PROBLEMAS com a comunicação da equipa de saúde em relação aos seguintes itens:

1. Alteração das datas dos exames, consultas ou tratamentos perante a sua impossibilidade de comparecer no dia agendado:
☐1 ☐2 ☐3 ☐4 ☐5 ☐N/A

2. Resolução de problemas que surgiram:
☐1 ☐2 ☐3 ☐4 ☐5 ☐N/A

3. Informação de tempo previsto para o seu exame, consulta ou tratamento:
☐1 ☐2 ☐3 ☐4 ☐5 ☐N/A

X- Classifique a sua SATISFAÇÃO GLOBAL em relação à resolução de problemas:
☐1 ☐2 ☐3 ☐4 ☐5

XI- Avalie a sua satisfação com o MATERIAL E APOIO na comunicação da equipa de saúde em relação aos seguintes itens:

1. E-mail fornecido (para contactar se necessitasse de alguma informação ou apoio):
☐1 ☐2 ☐3 ☐4 ☐5 ☐N/A

2. Relatório fornecido (com o que se passou no exame, consulta ou tratamento):
☐1 ☐2 ☐3 ☐4 ☐5 ☐N/A

3. Resumo em papel de como vai tomar a medicação:
☐1 ☐2 ☐3 ☐4 ☐5 ☐N/A

4. Plano de tratamento:
☐1 ☐2 ☐3 ☐4 ☐5 ☐N/A

XII- Classifique a sua SATISFAÇÃO GLOBAL em relação ao material de apoio:

☐1 ☐2 ☐3 ☐4 ☐5

Comentário que ache conveniente:

Obrigada pela colaboração.
